# Vagally-mediated heart block after myocardial infarction associated with plasticity of epicardial neurons controlling the atrioventricular node

**DOI:** 10.3389/fnsyn.2022.960458

**Published:** 2022-08-15

**Authors:** John D. Tompkins, Una Buckley, Siamak Salavatian, Kalyanam Shivkumar, Jeffrey L. Ardell

**Affiliations:** Neurocardiology Research Program of Excellence, Cardiac Arrhythmia Center, David Geffen School of Medicine, University of California, Los Angeles, Los Angeles, CA, United States

**Keywords:** intrinsic cardiac neurons, vagus nerve, electrophysiology, atrioventricular block, autonomic ganglion, Yorkshire pig

## Abstract

Imbalances in the opposing actions of sympathetic and parasympathetic nerves controlling the heart enhance risk for arrhythmia and sudden cardiac death after myocardial infarction (MI). Plasticity in peripheral neuron function may underlie the observed changes in cardiomotor nerve activity. We studied vagal control of the heart in pigs after chronic infarction of the left ventricle. Stimulation of the cervical vagus nerve produced greater bradycardic responses 8-weeks after MI. Recordings of epicardial electrocardiograms demonstrate increased severity and duration of atrioventricular (AV) block in MI-pigs during 20 Hz vagal stimulation. Intracellular voltage recordings from isolated neurons of the inferior vena cava-inferior left atrium (IVC-ILA) ganglionated plexus, a cluster of epicardial neurons receiving innervation from the vagus known to regulate the AV node, were used to assess plasticity of membrane and synaptic physiology of intrinsic cardiac neurons (ICNs) after MI. Changes to both passive and active membrane properties were observed, including more negative resting membrane potentials and greater input resistances in MI-pig ICNs, concomitant with a depression of neuronal excitability. Immunoreactivity to pituitary adenylate cyclase-activating polypeptide (PACAP), a cardiotropic peptide known to modulate cardiac neuron excitability, was localized to perineuronal varicosities surrounding pig IVC-ILA neurons. Exogenous application of PACAP increased excitability of control but not MI-ICNs. Stimulation (20 Hz) of interganglionic nerves in the *ex vivo* whole-mount preparations elicited slow excitatory postsynaptic potentials (sEPSPs) which persisted in hexamethonium (500 μM), but were blocked by atropine (1 μM), indicating muscarinic receptor-mediated inhibition of M-current. Extracellular application of 1 mM BaCl_2_ to inhibit M-current increased neuronal excitability. The muscarine-sensitive sEPSPs were observed more frequently and were of larger amplitude in IVC-ILA neurons from MI animals. In conclusion, we suggest the increased probability of muscarinic sEPSPs play a role in the potentiation of the vagus nerve mediated-slowing of AV nodal conduction following chronic MI. We identify both a novel role of a muscarinic sensitive current in the regulation of synaptic strength at ICNs projecting to the AV node, and demonstrate changes to both intrinsic plasticity and synaptic plasticity of IVC-ILA neurons which may contribute to greater risk for heart block and sudden cardiac death after MI.

## Introduction

Signaling by autonomic nerves at nodal cells, Purkinje fibers, and cardiac myocytes, controls sinus rhythm, atrioventricular conduction, and cardiac contractility (Ardell and Armour, 2016). Following ischemic injury to heart tissue, as occurs during myocardial infarction (MI), the well balanced and coordinated neural control of the heart is adversely remodeled—characterized by heightened sympathetic nerve and diminished parasympathetic nerve signaling (Fukuda et al., 2015; Salavatian et al., 2017). Imbalances in this autonomic control of cardiac function contribute to greater potential for ventricular arrhythmias and sudden cardiac death (Zipes and Rubart, 2006; Vaseghi and Shivkumar, 2008).

The heart receives bilateral innervation from sympathetic and parasympathetic nerves containing both sensory (afferent) and efferent (sympathetic or parasympathetic) neurons (Parsons, 2004; Ardell and Armour, 2016). Preganglionic cell bodies in the brainstem or intermediolateral cell column of the spinal cord project axons to peripheral neuron cell bodies of parasympathetic or sympathetic ganglia, respectively. Sympathetic preganglionic fibers synapse onto the postganglionic neurons of the stellate and middle cervical ganglia. Parasympathetic preganglionic axons project through the vagus to form synaptic connections with the intrinsic cardiac neurons (ICNs) distributed across multiple sites on the epicardial surface of the heart (Batulevicius et al., 2008). The ICNs also receive input from postganglionic sympathetic neurons, and some ICNs express enzymes for catecholamine synthesis or sensory neuropeptides (Smith, 1999; Richardson et al., 2003; Weihe et al., 2005; Hoard et al., 2008; Hoover et al., 2009a). A subpopulation of ICNs transduce sensory inputs arising from atrial and ventricular tissues (Ardell et al., 1991). A distributed network of autonomic nerves and ganglia on the heart regulate specific areas of cardiac control and make up the intrinsic cardiac nervous system (ICNS) (Ardell and Armour, 2016).

Days to weeks following MI, remodeling of the cardiac neural network becomes evident. This is a result of both direct and indirect effects on cardiac nerves. At the site of the infarct, regional loss of sympathetic nerve fibers (Dae et al., 1995), and hypersensitivity to catecholamines in the surviving myocardium contribute to the genesis of electrical instability in the ventricular myocardium (Barber et al., 1983; Cao et al., 2000; Ajijola et al., 2013). Paravertebral sympathetic neurons in the stellate ganglion show altered morphology and neurochemistry (Habecker et al., 2005; Ajijola et al., 2012, 2015; Nguyen et al., 2012). A sensitization of cardiac afferent nerves contributes to reflex augmentation of cardiac sympathetic nerve activity (Brown, 1967; Longhurst et al., 2001; Zahner et al., 2003; Bauer et al., 2006; Chan et al., 2015; Wang et al., 2017).

Cardiac parasympathetic dysfunction manifests as depressed baroreflex sensitivity and reduced heart rate variability (Cerati and Schwartz, 1991; Farrell et al., 1991). The changes are attributed, in part, to deficits in central processing of vagal sensory neurotransmission (Salavatian et al., 2017). Relatively few studies have investigated the peripheral neural circuits in the context of MI, with a paucity of data from large animals. In the pig, both ventricular repolarization times and ventricular acetylcholine concentrations were not different between control and MI animals after stimulation of the intact vagus nerve (Rajendran et al., 2016; Vaseghi et al., 2017). At the cellular level, hypertrophy of ICNs was observed along with greater expression of vasoactive intestinal polypeptide. The responsiveness of ICNs to cardiac stressors was also altered, as detected from extracellular neural recordings (Rajendran et al., 2016). In guinea pigs, 7–9 weeks after MI, ICNs show changes in expression of neuronal nitric oxide synthase, and intracellular microelectrode recordings established an altered responsiveness of ICNs to extracellularly applied histamine, as well as time dependent changes in synaptic efficacy (Hardwick et al., 2008, 2014). It is unclear how changes in ICN processing may reshape peripheral parasympathetic control as a thorough evaluation of vagus nerve mediated changes in cardiac performance was not determined in the above studies.

To test whether peripheral neural circuits involved in cardiac parasympathetic control remodel after MI, we evaluated the hearts responsiveness to cervical vagus nerve stimulation in anesthetized Yorkshire pigs, 6–8 weeks after chronic MI. The large animal model recapitulates cardiac neuroanatomy observed in human, and allows sophisticated measurements of cardiac function and careful control of MI lesions that are impractical in rodents and other small animals. Experiments were performed after transection of the vagus nerve to focus solely on efferent input to the heart (Ardell et al., 2015). After MI, anesthetized pigs showed greater susceptibility to atrioventricular (AV) block during high-frequency stimulation of the vagus. This hypersensitivity to parasympathetic input was subsequently found to be associated with altered neurotransmission at ICNs of the inferior vena cava-inferior left atrium (IVC-ILA) ganglionated plexus, a cluster of epicardial neurons associated with regulation of the AV node. The observed changes were consistent with facilitation of ganglionic cholinergic neurotransmission at IVC-ILA neurons from infarcted animals due to a greater probability of muscarinic slow depolarization. The work provides new evidence for intracardiac neuron remodeling following chronic MI, and shows that peripheral efferent parasympathetic neurotransmission is not only intact, but hyper-responsive after MI.

## Materials and methods

### Ethical approval

All whole animal research was conducted in accordance with the United States federal regulations as set forth in the Animal Welfare Act, the 2011 Guide for the Care and Use of Laboratory Animals (National Research Council Committee for the Update of the Guide for the Care & Use of Laboratory Animals, 2011), Public Health Service Policy for the Humane Care and Use of Laboratory Animals, as well as the University of California Los Angeles’ (UCLA’s) policies and procedures as set forth by the Animal Research Committee.

### Chronic infarction model

Male and female Yorkshire pigs (Premiere BioSource, CA, United States) (*n* = 15, 11 male, 4 female; age, 4–6 months) were sedated with midazolam (0.25–1 mg/kg IM) and ketamine (5–10 mcg/kg IM). Following intubation, general anesthesia was maintained with isoflurane (1–2% inhaled). Heart rate, arterial blood pressure, ventricular pressure and surface electrocardiograms were recorded. An 8-French sheath was placed in the right femoral artery. An Amplatz Super Stiff Guidewire with J-Tip (Boston Scientific, Marlborough, MA, United States) was used to guide the Amplatz left curve (AL2, Boston Scientific, Marlborough, MA United States) coronary guide catheter under fluoroscopy to the left main coronary artery. Contrast angiography was performed to visualize the left coronary anatomy. A balanced middleweight wire (Abott Vascular, Temecula, CA, United States) was directed into the LAD and a 3.0 × 20 mm 135 mm internal diameter over the wire angioplasty balloon (FoxCross PTA Catheter; Abbot Vascular, Temecula, CA United States) was positioned after the first diagonal. The angioplasty balloon was inflated to occlude flow after the first diagonal and 3–5 mL saline suspension of microspheres (Polybead, 90 μm diameter; Polysciences Inc., Warrington, PA, United States) injected through the central lumen of the angioplasty balloon catheter. Occlusion of the artery was confirmed by contrast angiography, and acute myocardial infarction was confirmed by the presence of ST-segment elevations in the limb and precordial leads. Premedication with lidocaine 2 mg/kg iv, amiodarone 1.5 mg/kg im, heparin 5,000 iu iv, and esmolol 1 mg/kg iv (if adequate hemodynamics) were administered. The animals were recovered and terminal procedures were performed 8 weeks after MI induction. The size and location of the infarct has been confirmed in previously published studies and has not been repeated here (Rajendran et al., 2016; Vaseghi et al., 2017). Approximately 13% of animals (2/15) were lost to sudden cardiac death in the initial days and weeks after MI.

### Hemodynamics and vagal stimulation in anesthetized whole animal

Control (*n* = 7; 5 male, 2 female) and MI (*n* = 6; 5 male, 1 female) Yorkshire pigs (age 6–8 months) were sedated with intramuscular telazol (4–6 mg/kg), intubated, and mechanically ventilated. General anesthesia was maintained with inhaled isoflurane (1.5–2.5%) and intravenous boluses of fentanyl (total: 10–30 μg/kg) during surgical preparation. A median sternotomy was performed to expose the heart. Continuous intravenous saline was infused through the femoral vein throughout experiments to maintain volume homeostasis. Arterial blood pressure was measured via a femoral arterial line. Heart rate (HR) was monitored by lead II ECG. Left ventricular systolic pressure was measured using a pressure monitoring pigtail catheter (5 Fr) inserted into the left ventricle (LV) via the left carotid artery and connected to a PCU-2000 pressure control system (Millar Instruments, Houston, TX). Arterial blood gas was tested hourly, and adjustments of ventilation and/or administration of sodium bicarbonate were made as necessary to maintain acid-base homeostasis. Hemodynamic data were acquired with a data acquisition system (Power1401, Cambridge Electronic Design, Cambridge, United Kingdom) and analyzed offline with Spike2 (Cambridge Electronic Design). Derived indexes included HR, aortic blood pressure, LV end-systolic pressure, maximum rate of change in LV pressure (LV dp/dt maximum), and minimum rate of change in LV pressure (LV dp/dt minimum). Offline analysis was used to determine the average response for each of the parameters at baseline and during vagus nerve stimulation (VNS).

Bipolar stimulating helical cuff electrodes (PerenniaFlex model 304, Cyberonics; Houston, TX) were placed around the right and left cervical vagus nerves, with the anodes positioned cephalad to the cathode. In 9 animals (*n* = 5 control, *n* = 4 MI), the cervical vagus nerves were transected bilaterally, 2–3 inches rostral of the stimulating electrodes, to eliminate centrally mediated responses that result from activation of centrally projecting vagal afferent fibers. In a pig model, this site has been shown to contain few if any cardiac sympathetic nerves (Dacey et al., 2022). A stimulator with a photoelectric constant-current isolation unit (models S88 and PSIU6, Grass Technologies, Warwick, RI) was used to deliver square pulses to the cuff electrodes. Bradycardia threshold was defined as the current required to produce a 20% decrease in HR at a frequency of 10 Hz, a pulse width of 5 ms, and a duration of 20 s. The effects of VNS on chronotropic, dromotropic, LV inotropic, and LV lusitropic function were evaluated over a range of currents (0.25–4.0 mA) at 2, 5, 10, and 20 Hz stimulation frequencies. VNS was performed for 20 s followed by a 5 min off-phase. This time period was sufficient for return of cardiac indexes to baseline values, with no degradation in the responses to VNS over the duration of the experiments. Experiments were completed on the right and left vagus nerves independently, with randomization of starting side and stimulation frequencies.

### Intracellular recordings from cardiac ganglion whole-mount preparations

Hearts were removed from animals (*n* = 10 controls; 8 male, 2 female; age 6–8 mo; *n* = 11 MIs; 8 male, 3 female; age 6–8 mo) deeply anesthetized with isoflurane (4–5%), and were placed in ice cold physiological salt solution (PSS) containing (in mM): 121 NaCl, 5.9 KCl, 1.2 NaH_2_PO_4_, 1.2 MgCl_2_, 25 NaHCO_3_, 2 CaCl_2_, 8 D-glucose; pH 7.4 maintained by 95% O_2_-5% CO_2_ aeration. A region of the dorsal epicardium located at the inferior vena cava, and inferior left atrium was isolated from the heart and pinned, epicardial surface down, to the Sylgard (Dow Corning) floor of a glass bottomed petri dish. The overlying endocardium was removed with fine forceps to reveal epicardial ganglia. The isolated preparations were continuously superfused (6–7 mL/min) with PSS maintained at 32 ± 2^°^C. Individual neurons, visualized with an upright microscope equipped with a 40x water immersion objective, were impaled using borosilicate glass microelectrodes filled with 2 M KCl (60–120 MΩ) or 2 M KCl + 1 M neurobiotin (Vector Labs, Burlingame, CA). Intracellular recordings from cardiac neurons followed methods described previously (Hanna et al., 2021). Membrane voltage was recorded using a Multiclamp 700B amplifier coupled with a Digidata 1550B data acquisition system and pCLAMP 10 software (Molecular Devices, Sunnyvale, CA). Depolarizing and hyperpolarizing currents were applied through the recording electrode to characterize neuronal membrane properties. Hyperpolarizing current steps (500 ms) of increasing amplitude were used to test membrane input resistance, and determine steady state current-voltage relationships. Graphs of neuronal excitability were made by plotting the number of action potentials generated by depolarizing current intensity (500 ms; Δ100 pA) (Tompkins et al., 2016).

### Immunostaining and confocal imaging of cardiac neurons

Cardiac neurons in the ganglion whole-mount preparations were immunostained following procedures reported previously (Jungen et al., 2019; Hanna et al., 2021). Ganglion whole-mounts were fixed in 4% paraformaldehyde, overnight, at 4^°^C. Tissue was rinsed (3 × 1 h wash) in 0.01 M phosphate-buffered saline (PBS), and stored in 0.01 M PBS with 0.02% sodium azide. For immunohistochemical staining, tissue was blocked in 0.01 M PBS, 0.02% sodium azide, 0.1% Triton X-100, and horse serum for 4 h at room temperature with agitation and then incubated in primary antibodies, in a solution of 0.01 M PBS, 0.02% sodium azide, and 0.1% Triton X-100, with agitation for two nights. The source and concentration of antibodies used were as follows: rabbit anti-PGP9.5 1:500 (Abcam, ab108986), sheep anti-TH 1:200 (Millipore Sigma, AB1542), mouse anti-PACAP 1:10 (MabJHH1; gift from Dr. Jan Fahrenkrug, Copenhagen, Denmark). The mouse monoclonal PACAP antibody has been previously validated (Hannibal et al., 1995). Preabsorbtion of the antibody with PACAP38 and PACAP27 (20 μg/mL) abolished all staining. After incubation with primaries, tissues were washed with a solution of 0.01 M PBS, 0.02% sodium azide every hour for 3 h then incubated in secondary antibodies, diluted in 0.01 M PBS with 0.1% Triton X-100 and 0.02% sodium azide, for two nights at room temperature with agitation. The following secondary antibodies were used: donkey anti-rabbit Cy3 (Jackson ImmunoResearch, 711-165-152, 1:200), donkey anti-sheep 488 (Jackson ImmunoResearch, 713-545-147, 1:200), donkey anti-mouse Cy3 (Jackson ImmunoResearch, 715-005-150, 1:200). Secondary staining with streptavidin conjugated ATTO-647N was used to visualize neurobiotin filled cells (1:500). Stained tissue was rinsed in 0.01 M PBS, 0.02% sodium azide every hour for 3 h and mounted on glass slides in anti-fade media (Citifluor, Electron Microscopy Sciences) and coverslipped.

Whole ganglia were imaged with confocal microscopes (either Leica SP5 or Zeiss 880) by tile-scanning in X, Y, and Z planes using either a 40X (Leica Plan-Apo, 1.25NA) or a 63X objective (Zeiss Plan-Apo, 1.4NA). Z-stack images were acquired at step sizes consistent with Nyquist sampling in relation to the numerical aperture of the objective. Three-dimensional scans of whole ganglion neuroanatomy were acquired with 10X objectives. Images were quantified using ImageJ to measure neuronal cross-sectional areas (measured at Z-plane containing nucleolus), and total cell counts per ganglia were quantified with ImageJ (FIJI distribution) (Schindelin et al., 2012).

### Statistics

Statistical analysis was performed using GraphPad Prism statistical software (version 9.2; La Jolla, CA). Data are presented as means ± SD. Means between two groups were compared with an unpaired *t*-test. Categorical data arranged into contingency tables were analyzed using Fisher’s exact test. Grouped data with two categorical variables and one continuous dependent variable were compared by two-way ANOVA and Šidák’s multiple comparisons test. Values were considered statistically significant at *P* < 0.05.

## Results

### Post-myocardial infarction hearts are susceptible to atrioventricular conduction block during stimulation of the left vagus nerve

**FIGURE 1:**
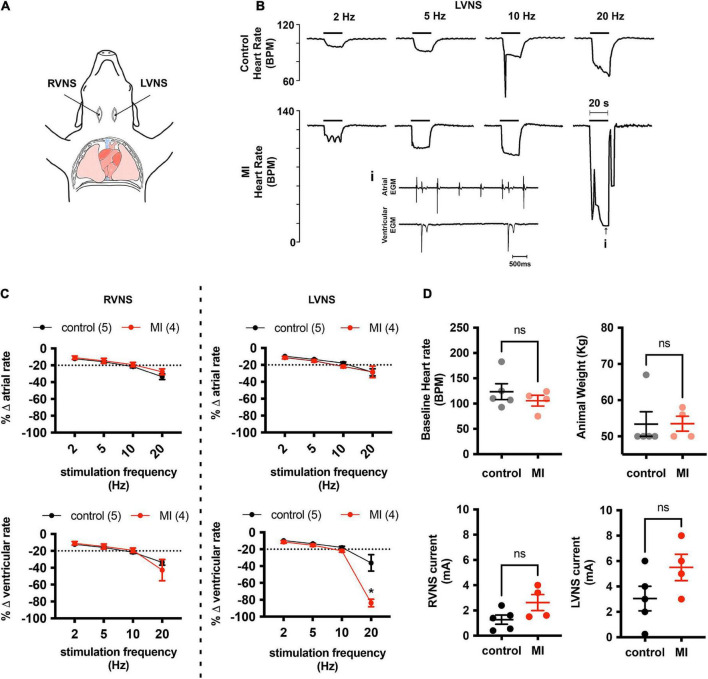
MI hearts are more susceptible to AV conduction block during stimulation of the left vagus nerve. **(A)** Cardiac responsiveness to cervical vagal stimulation was assessed in anesthetized, open chest pigs, after bilateral transection of the vagus nerves. RVNS, right vagus nerve stimulation; LVNS, left vagus nerve stimulation. **(B)** Example tracings of ventricular rate from a control (*top*) and MI (*bottom*) animal during stimulation of the left cervical vagus nerve (LVNS) at 5, 10, and 20 Hz. Note the augmented bradycardic response in MI animal due to AV block (*inset*) during 20 Hz stimulation. This was typical for all MI animals tested. **(C)** Summary changes in atrial and ventricular rate during stimulation of the right (RVNS) and left (LVNS) cervical vagus. In MI animals, 20 Hz LVNS stimulation produced a significantly greater decrease in ventricular rate due to propensity for AV node block. * Indicates *P* < 0.05, Šidák’s multiple comparisons test. **(D)** Baseline heart rate, animal weights, and current intensity for LVNS or RVNS were not different between groups.

At baseline, left ventricular systolic pressure (LVSP) and positive and negative peak changes in dP/dt were similar between control (LVSP 99 ± 25 mmHg; + dP/dt 1,376 ± 168 mmHg/s; −dP/dt −1426 ± 381 mmHg/s; *n* = 4) and MI pigs (LVSP 108 ± 34 mmHg, *P* = 0.66; + dP/dt 1,449 ± 555 mmHg/s, *P* = 0.81; −dP/dt −1735 ± 859 mmHg/s, *P* = 0.53; *n* = 4). Stimulation of the intact vagus in a subset of animals elicited biphasic responses, with a brief bradycardia followed by tachycardia. To selectively isolate stimulation of cardiac projecting vagal efferent fibers, the cervical vagus was transected and the distal nerve was stimulated. After bilateral transection of the cervical vagi, baseline heart rate was unchanged in both control (90 ± 21 vs. 105 ± 23 bpm, *P* = 0.38, *n* = 4) and MI animals (90 ± 21 vs. 105 ± 23 bpm, *P* = 0.35, *n* = 4). After an equilibration period of 20 min, the currents required to produce a 20% drop in heart rate were similar between animal treatment groups for both left-side and right-side vagal stimulation (LVNS 3.05 ± 2.15 mA control vs. 5.5 ± 2.08 mA MI, *P* = 0.13; RVNS 1.27 ± 0.82 mA control vs. 2.63 ± 1.27 mA MI, *P* = 0.09). Vagal stimulation elicited frequency dependent decreases in atrial and ventricular rate, with the greatest decreases observed after 20 Hz stimulation. At 20 Hz stimulation frequencies, ventricular rate slowed to 84 ± 9% of baseline in MI animals (*n* = 4), vs. 36 ± 22% in controls (*n* = 5), due largely to presence of AV block (Figure 1). Third degree heart block was observed in all MI animals (4/4) with 20 Hz stimulation, while in control animals, AV block was only observed in one of five animals. The maximum P-R interval in MI pigs during 20 Hz LVNS was 4.3 ± 2.8 s.

### Hypertrophy of inferior vena cava-inferior left atrium ganglion neurons after myocardial infarction

**FIGURE 2:**
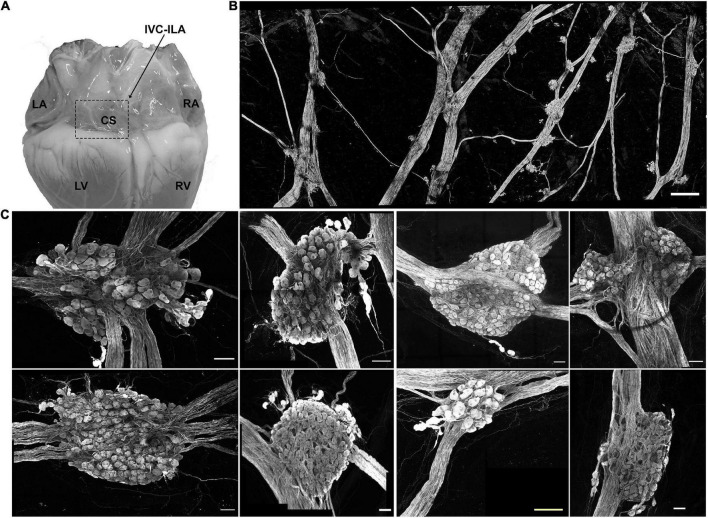
Neuroanatomy of the IVC-ILA ganglionated plexus. **(A)** Area of the IVC-ILA is circumscribed by the hatched line on a photograph of the dorsal surface of the porcine heart. LA, left atrium; RA, right atrium; CS, coronary sinus; LV, left ventricle; RV, right ventricle. **(B)** Stitched confocal image (10×) showing the network of interganglionic nerves and intrinsic cardiac ganglia within the boxed area in **(A)**. scale bar: 1 mm **(C)** Representative examples of intrinsic cardiac ganglia isolated from control (*left four panels*) and MI (*right four panels*) pig hearts. Scale bars: 100 μm.

**FIGURE 3:**
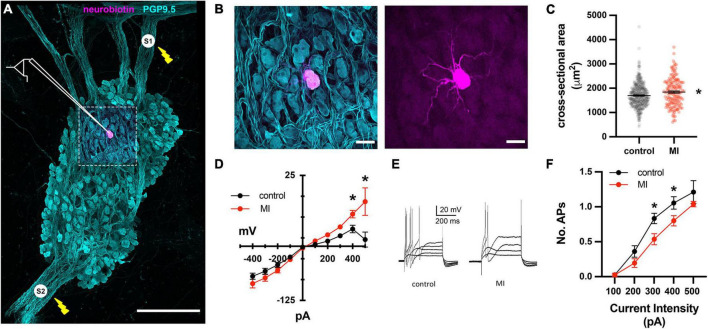
Depressed excitability of ICNs from MI animals shown by intracellular assessment of ICN membrane physiology. **(A)** Schematic overlay of the stimulation and recording configuration over a stitched confocal image (40×) of an individual intrinsic cardiac ganglion. Bipolar stimulating electrodes (*labeled S1 and S2*) were placed on interganglionic nerves. The magenta cell was backfilled with neurobiotin from the recording electrode. Scale bar is 500 μm. **(B)** Split images of boxed region in **(A)**, showing all cells (*left*) and single labeled cell (*right*). Scale bar 50 μm for both panels. **(C)** The cross-sectional areas of ICNs from pigs with MI were larger than controls. * Indicates *P* < 0.05, Fisher’s exact test. **(D)** The steady state membrane voltage in response to intracellularly injected current is plotted for MI and control ICNs. Input resistances were similar between groups but MI cells tended to have greater depolarizations in response to 400 and 500 pA currents. * Indicates *P* < 0.05, Šidák’s multiple comparisons test. **(E)** Membrane potential responses to long duration (500 ms), intracellular depolarizing current pulses from a control and MI neuron. Note that ICNs from control animals typically fired multiple action potentials in response to depolarization, while MI ICNs only fired a single action potential. **(F)** The reduced excitability of MI neurons is evident from the decreased slope of the excitability curve, plotting the number of evoked action potentials vs. the range of depolarizing current pulse amplitudes. * Indicates *P* < 0.05, Šidák’s multiple comparisons test.

In Yorkshire pigs, a network of epicardial nerves converge and traverse the atrioventricular septum at the ostium of the coronary sinus (Figures 2A,B). Along these interconnecting nerve bundles lies the IVC-ILA ganglionated plexus, located within the same transverse plane as the AV node. Small groupings (3–4) of neuronal somata lie along the epicardial nerves, while the majority of cell bodies reside in ganglia at the intersections of these nerves (Figure 2C). We counted 27–291 neurons per ganglion, among 20 ganglia (107 ± 85 neurons). Given each IVC-ILA ganglionated plexus contained approximately 40–60 ganglia, we estimate each neural plexus in this region contains approximately 4,000–6,000 neurons. The area of the neuronal cell bodies, in sections containing nuclei, was greater after MI (1,700 ± 24 vs. 1,841 ± 45 μm^2^; *P* = 0.0031) (Figure 3C).

### Basic membrane properties of the intrinsic cardiac neurons are unaffected by myocardial infarction though excitability is altered

To investigate whether the enhanced sensitivity to vagal stimulation after MI is due to changes in synaptic transmission at ICNs, intracellular recordings were made from IVC-ILA neurons in acutely isolated, ganglion whole-mount preparations (Figure 3A,B). The passive and active membrane properties of cells from MI or control animals are reported in Table 1. Resting membrane potential and input resistance values were greater in ICNs from hearts after MI. The action potential duration was also prolonged in these cells (Table 1).

**TABLE 1:** Active and passive membrane properties of porcine IVC-ILA ICNs (mean ± SD).

	Control	MI	P
RMP (mV)	−59 ± 5 (39)	−61 ± 4 (40)*	0.018
Input resistance (MΩ)	91 ± 36 (39)	116 ± 68 (40)*	0.045
Tau (ms)	22 ± 11 (39)	19 ± 8 (40)	0.146
AP amplitude (mV)	65 ± 10 (31)	65 ± 9 (32)	0.848
AP 1/2 width (ms)	2.1 ± 0.5 (31)	2.4 ± 0.7 (32)*	0.044
AHP amplitude (mV)	−13 ± 4 (33)	−15 ± 3.1 (20)	0.099
AHP duration (ms)	170 ± 81 (33)	189 ± 77 (20)	0.410

* Indicates P < 0.05, unpaired t-test.

Long duration depolarizing currents (500 ms) were given to evaluate membrane excitability. In control animals, depolarizing currents (100–500 pA) typically (19/24) elicited only a single spike, while multiple action potentials (2–3) were observed in 5/24 ICNs (Figures 3D–F). After MI, neuronal excitability was depressed significantly, as evidenced by a reduced slope of the excitability curve (Figure 3F). All post-MI ICNs tested (22/22) fired only a single action potential in response to intracellular depolarizing currents (Figure 3E). Although of insufficient resolution to precisely calculate rheobase, a greater magnitude current step (340 ± 88 vs. 258 ± 76 pA, *P* = 0.0022) was required to initiate an action potential in MI neurons.

### Intrinsic cardiac neuron responsiveness to pituitary adenylate cyclase-activating polypeptide application is reduced after myocardial infarction

**FIGURE 4:**
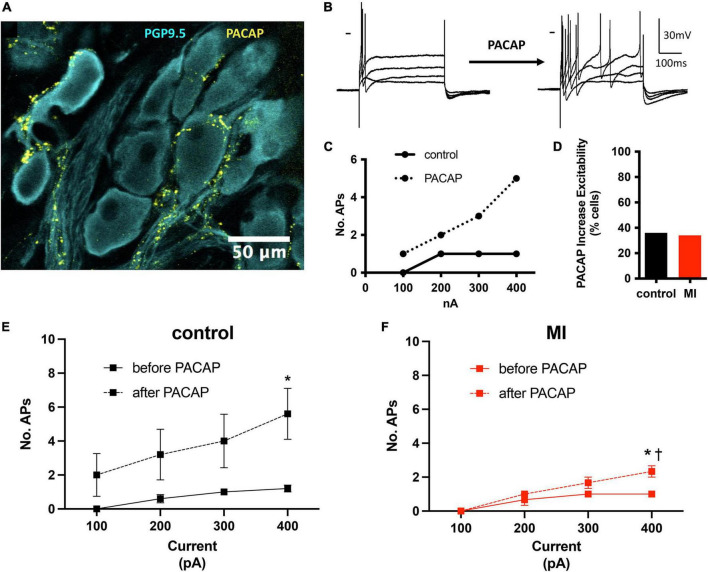
PACAP increased excitability of control and MI ICNs. **(A)** PACAP-immunoreactivity was localized to pericellular varicosities surrounding some, but not all ICNs. **(B)** Extracellular application of PACAP (20 nM) increased membrane excitability as evident by a greater number of action potentials in response to depolarizing current steps. Dash indicates 0 mV. Resting membrane potential = 58 mV. **(C)** The change in excitability for the response shown in B is plotted. Cells tended to fire only a single action potential before PACAP. After PACAP application this cell fired a maximum of five action potentials. **(D)** The percentage of ICNs from MI animals which fired a greater number of action potentials after PACAP application was similar to controls. **(E,F)** The summary excitability curves show mean changes in excitability before and after PACAP application for control ICNs **(E)** and MI ICNs **(F)**. The increase in excitability after PACAP was greater at control ICNs. * Indicates *P* < 0.05, Šidák’s multiple comparisons test. ^†^ Indicates *P* < 0.05 MI vs control, Šidák’s multiple comparisons test.

We have demonstrated previously that the neuropeptide PACAP is co-released with acetylcholine at vagal preganglionic nerve terminals in the guinea pig (Tompkins et al., 2007). Exogenous application of PACAP causes a long-lasting increase in neuronal excitability (Tompkins et al., 2018) and increases synaptic efficacy (Hoover et al., 2009b). Here, we have identified PACAP-like immunoreactive varicosities surrounding the porcine ICNs (Figure 4A). Bath application of PACAP (20 nM), increased porcine ICNs excitability (Figures 4B–F), demonstrating a role of this neuropeptide in regulation of membrane excitability at ICNs of large mammals. Approximately one third of all cells tested (control 5/13, MI 3/9) showed an increase in membrane excitability after PACAP, evident by a greater number of action potentials after depolarizing stimuli. In control cells, PACAP increased the number of action potentials elicited by maximum depolarizing currents (+400 pAs) from 1.2 ± 0.45 to 5.6 ± 3.36 (*P* = 0.02). In MI animals, the number of action potentials elicited by maximum depolarizing currents increased from 1 ± 0 to 2.33 ± 0.58 (*P* = 0.001). The increased number of action potentials, in response to maximum depolarizing stimuli, was greater in controls compared to MI after PACAP (5.6 ± 3.36 vs. 2.33 ± 0.58, *P* = 0.0014).

### Muscarinic receptor-mediated slow potentials are observed more frequently and are of greater amplitude at intrinsic cardiac neurons from hearts after myocardial infarction

Synaptically-evoked action potentials were produced by electrical stimulation of interganglionic nerves using concentric bipolar electrodes. Nerve evoked potentials were identified as either orthodromically or antidromically mediated by testing for inhibition of synaptically evoked potentials with hexamethonium (500 μM), a ganglionic nicotinic receptor antagonist. In ganglia isolated from both control and MI pigs, both antidromic and orthodromic action potentials were observed. Approximately half of the orthodromically-mediated potentials were subthreshold for generation of an action potential at control (18/35) and MI (15/31) neurons. Coactivation of two subthreshold inputs, in 3 control and 9 MI preparations, elicited a suprathreshold EPSPs (sufficient for initiation of an action potential) demonstrating convergence of synaptic inputs at some intracardiac neurons.

**FIGURE 5:**
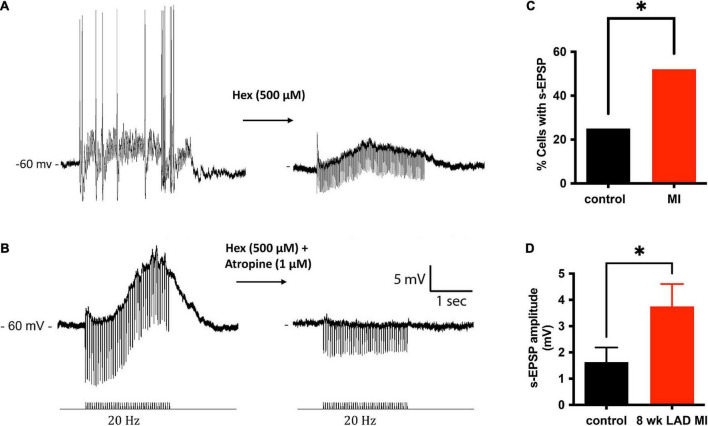
Muscarinic slow potentials are more prevalent and of greater amplitude after MI. **(A)** Representative tracings of a slow-EPSPs evoked from one cell before and after administration of hexamethonium (Hex). Hex blocked fast-synaptic potentials elicited by acetylcholine activation of ganglion nicotinic receptors. In the presence of Hex, the slow-EPSP could be elicited reproducibly, as long as the interval between trains was greater than 200 ms. **(B)** In a separate cell, in the presence of Hex, 20 Hz stimulation of an interganglionic nerve bundle caused a slow depolarization (∼13 mV) of the postsynaptic membrane. The slow depolarization was inhibited by application of the muscarinic receptor inhibitor atropine. **(C)** A significantly greater fraction of MI ICNs responded with muscarinic s-EPSPs. **(D)** In addition to being observed more frequently, the amplitudes of the s-EPSPs were greater in MI ICNs.

**FIGURE 6:**
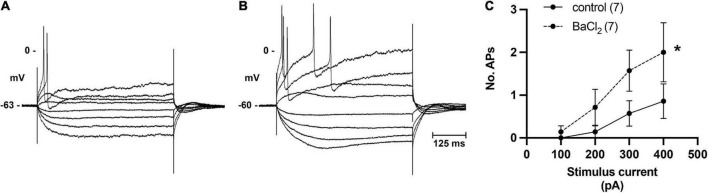
Inhibition of M-current by bath application of BaCl_2_ identifies role of M-current in regulation of pig ICN excitability. **(A,B)** Membrane potential tracing showing a typical response from a control ICN in control PSS and after extracellular addition of BaCl_2_ (1 mM). Barium application increased membrane excitability, evident by a greater number of action potentials in response to membrane depolarization. **(C)** In total (*n* = 7), barium caused a modest but significant change in the excitability of the cardiac neuron membrane.

In addition to single pulses, trains of stimuli at 5, 10, and 20 Hz frequencies for 5 s were also applied. Slow excitatory post-synaptic potentials (sEPSPs) were observed during 20 Hz stimulation. The sEPSPs were more easily discernable and could accurately be measured after hexamethonium (Figure 5A). At an interval ≥200 s, the slow EPSP was repeatedly reproduced in the same neuron. Given the reproducible nature of the response, and the known presence of M-current in cardiac neurons (Huang et al., 1993; Cuevas et al., 1997), atropine was administered to determine if the sEPSPs were mediated by muscarinic receptors. Five minutes after application, atropine completely blocked the nerve evoked sEPSPs in 5 of 5 cells tested (Figure 5B). Extracellular application of the M-current antagonist BaCl_2_ (1 mM), also increased excitability of ICNs (Figure 6), further supporting a role for M-current in regulation of membrane excitability of porcine ICNs.

The muscarinic slow depolarizations were observed in a greater number of ICNs from MI animals (12/23 vs. 7/28, *P* = 0.0438; Figure 5C,D). The mean change in membrane potential during 20 Hz stimulation, for all cells tested, was greater at ICNs from MI animals [3.75 ± 4.1 mV (*n* = 23) vs. 1.63 ± 2.9 mV (*n* = 28), *P* = 0.0363; Figure 5D]. In cells tested with hexamethonium, the hexamethonium resistant, atropine sensitive depolarizations to be greater at ICNs isolated from MI animals [7.57 ± 3.4 mV (*n* = 5)] compared to the control ICNs [4.9 ± 2.4 mV (*n* = 6), *P* = 0.1597].

## Discussion

We identify greater slowing of ventricular rate in response to high frequency stimulation of the left vagus nerve, 8 weeks after chronic occlusion of the distal LAD. Intracellular recordings from neurons within the IVC-ILA ganglionated plexus, a network of epicardial ganglia supplying innervation to the AV node, indicate the augmentation in the parasympathetic evoked bradycardia is due, in part, to remodeling of ganglionic neurotransmission at this site. After MI, porcine ICNs show depressed membrane excitability and hyperpolarized membrane potentials. Immunostaining reveals a pericellular network of PACAP-IR varicosities; however, exogenous application of PACAP had a greater effect at control vs MI ICNs. Despite the depressed excitability, high frequency stimulation of interganglionic nerves elicits muscarinic-mediated slow potentials more frequently in MI hearts. The atropine sensitive slow potentials are likely mediated by inhibition of M-current which acts to suppress membrane excitability. Extracellular application of barium to inhibit M-current increased the excitability of the IVC-ILA neurons. We postulate that the augmented bradycardia and greater propensity for AV block during high frequency stimulation of the left vagus are due to potentiation of ganglionic neurotransmission within epicardial ganglia as a result of a greater probability for muscarinic receptor mediated depolarization.

Our findings further support the concept of the epicardial ganglia forming an integrative intrinsic cardiac nervous system (ICNS) intimately involved in the local control of cardiac function (Smith, 1999; Parsons, 2004; Ardell and Armour, 2016). Within the peripheral efferent ganglia lies potential for modulation of synaptic strength, both at synapses within the ganglia on the heart and the thoracic ganglia. Here, and in a recent publication (Hanna et al., 2021), we show that neurotransmission within the porcine epicardial ganglia is not obligatory; rather, there is a convergence of subthreshold cholinergic synaptic inputs arriving from independent nerve bundles that summate on the postsynaptic membrane. Dye backfilling of the pig IVC-ILA neurons reveals complex dendritic arbors, contrasting the morphology of ICNs from smaller mammals, which have either very short (rat; Richardson et al., 2003) or no dendrites (mouse; Tompkins unpublished). In addition to cholinergic input, porcine ICNs are also surrounded by varicosities immunoreactive for PACAP based upon a validated monoclonal antibody recognizing PACAP-27 and PACAP-38 across multiple species (Hannibal, 2002). As observed in the guinea pig, PACAP increases the excitability of porcine ICNs. In the guinea pig, the PACAP effect is mediated by PAC1 receptors coupled to intracellular signaling cascades regulating multiple ionic conductances which support repetitive spiking in response to membrane depolarization (May et al., 2021). The observation that ICNs from control but not MI animals show a change in excitability after PACAP application, indicates that cardiac neurons from MI animals may be deficient in either the receptors for PACAP, the underlying signal transduction mechanisms, or a change in the baseline membrane physiology (i.e., greater expression of M-current).

Ganglionic neurotransmission is governed by mechanisms affecting both synaptic strength and membrane excitability. In rats, both H-current and M-current contribute to excitability of ICNs (Cuevas et al., 1997). M-current is a non-inactivating potassium current triggered by membrane depolarization and is known to modulate excitability and synaptic efficacy at autonomic neurons, as originally discovered from bullfrog sympathetic ganglia (Brown and Adams, 1980; Brown, 2020) and later in mammals including rat intracardiac ganglia (Cuevas et al., 1997). Inhibition of M-current by extracellular application of barium depolarizes the rat intracardiac neurons (Whyte et al., 2009). The findings of a depressed excitability and a more hyperpolarized resting membrane potential of ICNs from infarcted hearts are consistent with an augmentation of M-current in these cells. Release of this hyperpolarizing “brake” through M_1_-receptor dependent inhibition of KCNQ2/3 is known to cause membrane depolarization and increase neuronal excitability (Hille et al., 2014). The inhibition of M-current by extracellular application of barium in our experiments supports a role for M-current in the regulation of porcine ICN excitability. Intriguingly, reactive oxygen species (ROS) known to be released during transient ischemia induce M-current at rat intrinsic cardiac ganglion neurons (Whyte et al., 2009). In addition to the voltage dependent potassium channels, voltage dependent calcium channels also regulate neuronal excitability and neurotransmitter release. Decreased N-type Ca^2+^ current has been associated with depressed excitability of rat epicardial neurons (Tu et al., 2014; Zhang et al., 2018). Ca^2+^-activated potassium channels also contribute to the repolarization phase of the action potential, and neuronal excitability can be increased following a decrease in the AHP. We observed no differences in the AHPs of control or MI neurons, indicating the change in excitability between groups was not due to differences in repolarization time.

Modulation of ICN membrane excitability can change the efficacy of synaptic transmission by altering the probability of spike generation. In the guinea pig, increasing the membrane excitability of ICNs by application of the PAC1 receptor agonist maxadilan amplifies the vagally-mediated bradycardia (Hoover et al., 2009b). Changes in the efficacy of ganglionic neurotransmission on the epicardial surface of the heart affects the pattern of action potentials propagated to neuroeffector junctions throughout the heart, including vascular smooth muscle cells, nodal cells, and the myocardium. Altered ganglionic neurotransmission at epicardial ganglia can increase the potential for cardiac arrhythmias. In spontaneously hypertensive rats, a combination of increased density of synaptic boutons at ICNs, greater spontaneous release of acetylcholine and increased excitability of epicardial neurons contribute to the substrate for atrial fibrillation (Ashton et al., 2020). A decreased excitability of ICNs, due to a reduction in N-type Ca^2+^ channels, correlates with reduced parasympathetic control of ventricular myocardium in rats with heart failure resulting in greater susceptibility to ventricular arrhythmias (Zhang et al., 2018). ICN remodeling after MI has been observed in guinea pig (Hardwick et al., 2008, 2014) and Yorkshire pigs (Rajendran et al., 2016), but the correlation with altered parasympathetic nerve function was not tested. Here we provide the first intracellular characterization of Yorkshire pig ICNs after MI and demonstrate these cellular changes contribute to altered parasympathetic neural control of the heart. Additional work is required to determine if these changes increase potential for arrhythmias in this model.

Several limitations exist in regards to the conclusions drawn from this study. First, the ICNS exhibits regional specification in that ganglionated plexuses (GPs) located in specific epicardial regions innervate specific cardiac tissues (Cardinal et al., 2009). For example, the right atrial ganglionated plexus (RAGP) contains neurons projecting to the sinoatrial node, as evidenced by retrograde tracer and ganglion ablation experiments (Hanna et al., 2021), while the IVC-ILA GP supplies cholinergic innervation to the AV node (Ardell and Randall, 1986; Randall et al., 1986; Fee et al., 1987). Although target specificity is observed within each GP, overlap of innervation targets also occurs (Hanna et al., 2021). We focused on the IVC-ILA given its association with the AV node, but cannot be certain that the cells recorded from project to the AV node. We also cannot extrapolate these findings to other GPs (i.e., RAGP), as the effects of MI may be different at each site. For example, an infarct within a different region of the heart (e.g., right ventricle) might result in a different pattern of neuronal remodeling. We did not, however, observe any difference in right atrial rate in response to right or left-side VNS, indicating synaptic efficacy of RAGP neurons were not altered by the chronic infarction of the distal LAD. A second point is that epicardial ganglia contain mixed populations of neurons, which can be described as either cholinergic, adrenergic or nitrergic based on immunohistochemical staining (Parsons, 2004). More recently, identification of cell types based on single cell transcriptomic analysis has been presented (Hanna et al., 2021); however, the correlation with functional specificity or innervation target is still lacking. We randomly targeted IVC-ILA neurons for intracellular recording. The neurochemistry and innervation targets of these cells was not determined, therefor, the possibility of a sampling bias between cohorts exists. Lastly, we did not inhibit ganglionic muscarinic receptors, or modulate M-current, *in vivo* due to the broad distribution of these receptors and channels within other cardiac tissues. Also, only current clamp studies were performed so M-current was not quantified at IVC-ILA neurons between groups. Additional experimentation is required to more fully elucidate the contribution of the muscarinic-slow depolarization and regulation of ICN membrane excitability by M-current in the porcine model.

In summary, we provide evidence for neural plasticity within the ICNS after chronic MI in the Yorkshire pig. The changes in neuronal membrane properties include a depressed membrane excitability with greater probability for muscarinic receptor-mediated slow depolarizations during high frequency stimulation. The remodeling of the IVC-ILA GP neurons affects preganglionic parasympathetic nerve evoked bradycardia due to the action of these neurons projecting to the AV node. Despite the depressed membrane excitability at rest, the greater probability of post-synaptic muscarinic slow depolarizations following high frequency preganglionic nerve activity would increase synaptic efficacy, due both to membrane depolarization and inhibition of M-current, causing greater post-synaptic frequency of action potentials, greater acetylcholine release at the AV node resulting in a slowing, or block, of impulse conduction to the ventricles. The cause for the observed changes in ICN membrane properties after chronic MI are unknown. Plasticity in ICN function may result from changes in neuronal activity (e.g., activity dependent neuronal adaptation), circulating factors released at ischemic or border zone regions (e.g., ROS), or homeostatic response mechanisms related to changes in cardiovascular hemodynamics (e.g., renin-angiotensin-aldosterone system and/or natriuretic peptides). It is unlikely that the epicardial neurons are directly impacted by ischemia, as the ischemic zone (apex and distal ventricular septal wall) is distal to the IVC-ILA GP. Additional experimental work is required to more fully elucidate the ionic mechanisms regulating ganglionic neurotransmission in the porcine model and to correlate these changes with increased arrhythmia susceptibility after MI. Understanding the cellular mechanisms underlying both synaptic and innate plasticity of these epicardial neurons is fundamental to understanding the integrative role of this neural circuitry to cardiac homeostasis.

## Data availability statement

The datasets presented in this study can be found in online repositories. The names of the repository/repositories and accession number(s) can be found below: “National Institutes of Health, Common Fund, Stimulating Peripheral Activity to Relieve Conditions (SPARC) Program Data Resource Center: https://sparc.science.”

## Ethics statement

The animal study was reviewed and approved by University of California—Los Angeles Institutional Animal Care and Use Committee.

## Author contributions

JT designed *in vivo* and *in vitro* studies, with assistance from JA and KS for *in vivo* work. JT, UB, and SS collected and analyzed the data. JT and JA interpreted the data. JT wrote the manuscript with contributions from UB, JA, and KS. All authors approved the final version.

## Abbreviations

MI: myocardial infarction
ICN: intrinsic cardiac neuron
VNS: vagus nerve stimulation
PACAP: pituitary adenylate cyclase activating polypeptide
EPSP: excitatory post-synaptic potential
IVC-ILA: inferior vena cava–inferior left atrium
AV: atrioventricular
PBS: phosphate-buffered saline
LVSP: left ventricular systolic pressure.

## References

[B1] Ajijola O. A. Wisco J. J. Lambert H. W. Mahajan A. Stark E. Fishbein M. C. (2012). Extracardiac neural remodeling in humans with cardiomyopathy. *Circ. Arrhythm. Electrophysiol.* 5 1010–1116. 10.1161/CIRCEP.112.972836 22923270 PMC3529182

[B2] Ajijola O. A. Yagishita D. Patel K. J. Vaseghi M. Zhou W. Yamakawa K. (2013). Focal myocardial infarction induces global remodeling of cardiac sympathetic innervation: Neural remodeling in a spatial context. *Am. J. Physiol. Heart Circ. Physiol.* 305 H1031–H1040. 10.1152/ajpheart.00434.2013 23893167 PMC3798751

[B3] Ajijola O. A. Yagishita D. Reddy N. K. Yamakawa K. Vaseghi M. Downs A. M. (2015). Remodeling of stellate ganglion neurons after spatially targeted myocardial infarction: Neuropeptide and morphologic changes. *Heart Rhythm* 12 1027–1035. 10.1016/j.hrthm.2015.01.045 25640636 PMC4411181

[B4] Ardell J. L. Armour J. A. (2016). Neurocardiology: Structure-Based Function. *Compr. Physiol.* 6 1635–1653. 10.1002/cphy.c150046 27783854

[B5] Ardell J. L. Butler C. K. Smith F. M. Hopkins D. A. Armour J. A. (1991). Activity of in vivo atrial and ventricular neurons in chronically decentralized canine hearts. *Am. J. Physiol.* 260 H713–H721. 10.1152/ajpheart.1991.260.3.H713 2000967

[B6] Ardell J. L. Rajendran P. S. Nier H. A. KenKnight B. H. Armour J. A. (2015). Central-peripheral neural network interactions evoked by vagus nerve stimulation: Functional consequences on control of cardiac function. *Am. J. Physiol. Heart Circ. Physiol.* 309 H1740–H1752. 10.1152/ajpheart.00557.2015 26371171 PMC4666982

[B7] Ardell J. L. Randall W. C. (1986). Selective vagal innervation of sinoatrial and atrioventricular nodes in canine heart. *Am. J. Physiol.* 251 H764–H773. 10.1152/ajpheart.1986.251.4.H764 3021001

[B8] Ashton J. L. Argent L. Smith J. E. G. Jin S. Sands G. B. Smaill B. H. (2020). Evidence of structural and functional plasticity occurring within the intracardiac nervous system of spontaneously hypertensive rats. *Am. J. Physiol. Heart Circ. Physiol.* 318 H1387–H1400. 10.1152/ajpheart.00020.2020 32357112

[B9] Barber M. J. Mueller T. M. Henry D. P. Felten S. Y. Zipes D. P. (1983). Transmural myocardial infarction in the dog produces sympathectomy in noninfarcted myocardium. *Circulation* 67 787–796. 10.1161/01.CIR.67.4.787 6825234

[B10] Batulevicius D. Skripka V. Pauziene N. Pauza D. H. (2008). Topography of the porcine epicardiac nerve plexus as revealed by histochemistry for acetylcholinesterase. *Auton. Neurosci.* 138 64–75. 10.1016/j.autneu.2007.10.005 18063424

[B11] Bauer A. Kantelhardt J. W. Barthel P. Schneider R. Makikallio T. Ulm K. (2006). Deceleration capacity of heart rate as a predictor of mortality after myocardial infarction: Cohort study. *Lancet* 367 1674–1681. 10.1016/S0140-6736(06)68735-7 16714188

[B12] Brown A. M. (1967). Excitation of afferent cardiac sympathetic nerve fibres during myocardial ischaemia. *J. Physiol.* 190 35–53. 10.1113/jphysiol.1967.sp008191 6038025 PMC1365402

[B13] Brown D. A. (2020). Neurons, Receptors, and Channels. *Annu. Rev. Pharmacol. Toxicol.* 60 9–30. 10.1146/annurev-pharmtox-010919-023755 31914894

[B14] Brown D. A. Adams P. R. (1980). Muscarinic suppression of a novel voltage-sensitive K+ current in a vertebrate neurone. *Nature* 283 673–676. 10.1038/283673a0 6965523

[B15] Cao J. M. Fishbein M. C. Han J. B. Lai W. W. Lai A. C. Wu T. J. (2000). Relationship between regional cardiac hyperinnervation and ventricular arrhythmia. *Circulation* 101 1960–1969. 10.1161/01.CIR.101.16.1960 10779463

[B16] Cardinal R. Page P. Vermeulen M. Ardell J. L. Armour J. A. (2009). Spatially divergent cardiac responses to nicotinic stimulation of ganglionated plexus neurons in the canine heart. *Auton. Neurosci.* 145 55–62. 10.1016/j.autneu.2008.11.007 19071069

[B17] Cerati D. Schwartz P. J. (1991). Single cardiac vagal fiber activity, acute myocardial ischemia, and risk for sudden death. *Circ. Res.* 69 1389–1401. 10.1161/01.RES.69.5.1389 1934362

[B18] Chan Y. H. Tsai W. C. Shen C. Han S. Chen L. S. Lin S. F. (2015). Subcutaneous nerve activity is more accurate than heart rate variability in estimating cardiac sympathetic tone in ambulatory dogs with myocardial infarction. *Heart Rhythm* 12 1619–1627. 10.1016/j.hrthm.2015.03.025 25778433 PMC4485600

[B19] Cuevas J. Harper A. A. Trequattrini C. Adams D. J. (1997). Passive and active membrane properties of isolated rat intracardiac neurons: Regulation by H- and M-currents. *J. Neurophysiol.* 78 1890–1902. 10.1152/jn.1997.78.4.1890 9325358

[B20] Dacey M. Salahudeen O. Swid M. A. Carlson C. Shivkumar K. Ardell J. L. (2022). Structural and function organization of intrathoracic extracardiac autonomic projections to the porcine heart: Implications for targeted neuromodulation therapy. *Heart Rhythm* 19 975–983. 10.1016/j.hrthm.2022.01.033 35124232

[B21] Dae M. W. O’Connell J. W. Botvinick E. H. Chin M. C. (1995). Acute and chronic effects of transient myocardial ischemia on sympathetic nerve activity, density, and norepinephrine content. *Cardiovasc. Res.* 30 270–280. 10.1016/S0008-6363(95)00039-9 7585815

[B22] Farrell T. G. Paul V. Cripps T. R. Malik M. Bennett E. D. Ward D. (1991). Baroreflex sensitivity and electrophysiological correlates in patients after acute myocardial infarction. *Circulation* 83 945–952. 10.1161/01.CIR.83.3.945 1999042

[B23] Fee J. D. Randall W. C. Wurster R. D. Ardell J. L. (1987). Selective ganglionic blockade of vagal inputs to sinoatrial and/or atrioventricular regions. *J. Pharmacol. Exp. Ther.* 242 1006–1012. 2888868

[B24] Fukuda K. Kanazawa H. Aizawa Y. Ardell J. L. Shivkumar K. (2015). Cardiac Innervation and Sudden Cardiac Death. *Circul. Res.* 116 2005–2019. 10.1161/CIRCRESAHA.116.304679 26044253 PMC4465108

[B25] Habecker B. A. Gritman K. R. Willison B. D. Van Winkle D. M. (2005). Myocardial infarction stimulates galanin expression in cardiac sympathetic neurons. *Neuropeptides* 39 89–95. 10.1016/j.npep.2004.11.003 15752542

[B26] Hanna P. Dacey M. J. Brennan J. Moss A. Robbins S. Achanta S. (2021). Innervation and Neuronal Control of the Mammalian Sinoatrial Node a Comprehensive Atlas. *Circul. Res.* 128 1279–1296. 10.1161/CIRCRESAHA.120.318458 33629877 PMC8284939

[B27] Hannibal J. (2002). Pituitary adenylate cyclase-activating peptide in the rat central nervous system: An immunohistochemical and in situ hybridization study. *J. Comp. Neurol.* 453 389–417. 10.1002/cne.10418 12389210

[B28] Hannibal J. Mikkelsen J. D. Clausen H. Holst J. J. Wulff B. S. Fahrenkrug J. (1995). Gene expression of pituitary adenylate cyclase activating polypeptide (PACAP) in the rat hypothalamus. *Regul. Pept.* 55 133–148. 10.1016/0167-0115(94)00099-J 7754101

[B29] Hardwick J. C. Ryan S. E. Beaumont E. Ardell J. L. Southerland E. M. (2014). Dynamic remodeling of the guinea pig intrinsic cardiac plexus induced by chronic myocardial infarction. *Auton. Neurosci.* 181 4–12. 10.1016/j.autneu.2013.10.008 24220238 PMC3944072

[B30] Hardwick J. C. Southerland E. M. Ardell J. L. (2008). Chronic myocardial infarction induces phenotypic and functional remodeling in the guinea pig cardiac plexus. *Am. J. Physiol. Regul. Integr. Comp. Physiol.* 295 R1926–R1933. 10.1152/ajpregu.90306.2008 18832084 PMC2685292

[B31] Hille B. Dickson E. Kruse M. Falkenburger B. (2014). Dynamic metabolic control of an ion channel. *Prog. Mol. Biol. Transl. Sci.* 123 219–247. 10.1016/B978-0-12-397897-4.00008-5 24560147

[B32] Hoard J. L. Hoover D. B. Mabe A. M. Blakely R. D. Feng N. Paolocci N. (2008). Cholinergic neurons of mouse intrinsic cardiac ganglia contain noradrenergic enzymes, norepinephrine transporters, and the neurotrophin receptors tropomyosin-related kinase A and p75. *Neuroscience* 156 129–142. 10.1016/j.neuroscience.2008.06.063 18674600 PMC2640831

[B33] Hoover D. B. Isaacs E. R. Jacques F. Hoard J. L. Page P. Armour J. A. (2009a). Localization of multiple neurotransmitters in surgically derived specimens of human atrial ganglia. *Neuroscience* 164 1170–1179. 10.1016/j.neuroscience.2009.09.001 19747529

[B34] Hoover D. B. Tompkins J. D. Parsons R. L. (2009b). Differential activation of guinea pig intrinsic cardiac neurons by the PAC1 agonists maxadilan and pituitary adenylate cyclase-activating polypeptide 27 (PACAP27). *J. Pharmacol. Exp. Ther.* 331 197–203. 10.1124/jpet.109.155747 19602551 PMC2766232

[B35] Huang M. H. Smith F. M. Armour J. A. (1993). Modulation of in situ canine intrinsic cardiac neuronal activity by nicotinic, muscarinic, and beta-adrenergic agonists. *Am. J. Physiol.* 265 R659–R669. 10.1152/ajpregu.1993.265.3.R659 8105703

[B36] Jungen C. Scherschel K. Flenner F. Jee H. Rajendran P. De Jong K. A. (2019). Increased arrhythmia susceptibility in type 2 diabetic mice related to dysregulation of ventricular sympathetic innervation. *Am. J. Physiol. Heart Circ. Physiol.* 317 H1328–H1341. 10.1152/ajpheart.00249.2019 31625779 PMC6962614

[B37] Longhurst J. C. Tjen A. L. S. C. Fu L. W. (2001). Cardiac sympathetic afferent activation provoked by myocardial ischemia and reperfusion. Mechanisms and reflexes. *Ann. N. Y. Acad. Sci.* 940 74–95. 10.1111/j.1749-6632.2001.tb03668.x 11458709

[B38] May V. Johnson G. C. Hammack S. E. Braas K. M. Parsons R. L. (2021). PAC1 Receptor Internalization and Endosomal MEK/ERK Activation Is Essential for PACAP-Mediated Neuronal Excitability. *J. Mol. Neurosci.* 71 1536–1542. 10.1007/s12031-021-01821-x 33675454 PMC8450765

[B39] National Research Council (US) Committee for the Update of the Guide for the Care and Use of Laboratory Animals (2011). *Guide for the care and use of laboratory animals.* 8th ed. Washington, DC: National Academies Press (US). 10.17226/12910 21595115

[B40] Nguyen B. L. Li H. Fishbein M. C. Lin S. F. Gaudio C. Chen P. S. (2012). Acute myocardial infarction induces bilateral stellate ganglia neural remodeling in rabbits. *Cardiovasc. Pathol.* 21 143–148. 10.1016/j.carpath.2011.08.001 22001051 PMC3267867

[B41] Parsons R. L. (2004). “Mammalian cardiac ganglia as local integration centers: Histochemical and electrophysiological evidence,” in *Neural mechanisms of cardiovascular regulation*, eds Dun N. J. Machado B. H. Pilowsky P. M. (Boston: Kluwer Academic Publishers), 335–356. 10.1007/978-1-4419-9054-9_15

[B42] Rajendran P. S. Nakamura K. Ajijola O. A. Vaseghi M. Armour J. A. Ardell J. L. (2016). Myocardial infarction induces structural and functional remodelling of the intrinsic cardiac nervous system. *J. Physiol.* 594 321–341. 10.1113/JP271165 26572244 PMC4713729

[B43] Randall W. C. Ardell J. L. Calderwood D. Milosavljevic M. Goyal S. C. (1986). Parasympathetic ganglia innervating the canine atrioventricular nodal region. *J. Auton. Nerv. Syst.* 16 311–323. 10.1016/0165-1838(86)90036-6 3745782

[B44] Richardson R. J. Grkovic I. Anderson C. R. (2003). Immunohistochemical analysis of intracardiac ganglia of the rat heart. *Cell Tissue Res.* 314 337–350. 10.1007/s00441-003-0805-2 14523644

[B45] Salavatian S. Yamaguchi N. Hamon D. Fishbein M. C. Ardell J. L. Shivkumar K. (2017). Myocardial Infarction Causes Both Structural and Functional Remodeling in Cardiac Neurons of the Inferior Vagal (Nodose) Ganglia: Implications for Mechanisms Behind Parasympathetic Withdrawal in Heart Disease. *Circulation* 136:A17355.

[B46] Schindelin J. Arganda-Carreras I. Frise E. Kaynig V. Longair M. Pietzsch T. (2012). Fiji: An open-source platform for biological-image analysis. *Nat. Methods* 9 676–682. 10.1038/nmeth.2019 22743772 PMC3855844

[B47] Smith F. M. (1999). Extrinsic inputs to intrinsic neurons in the porcine heart in vitro. *Am. J. Physiol.* 276 R455–R467. 10.1152/ajpregu.1999.276.2.R455 9950925

[B48] Tompkins J. D. Ardell J. L. Hoover D. B. Parsons R. L. (2007). Neurally released pituitary adenylate cyclase-activating polypeptide enhances guinea pig intrinsic cardiac neurone excitability. *J. Physiol.* 582 87–93. 10.1113/jphysiol.2007.134965 17495034 PMC2075297

[B49] Tompkins J. D. Clason T. A. Buttolph T. R. Girard B. M. Linden A. K. Hardwick J. C. (2018). Src family kinase inhibitors blunt PACAP-induced PAC1 receptor endocytosis, phosphorylation of ERK, and the increase in cardiac neuron excitability. *Am. J. Physiol. Cell Physiol.* 314 C233–C241. 10.1152/ajpcell.00223.2017 29141923 PMC5866440

[B50] Tompkins J. D. Clason T. A. Hardwick J. C. Girard B. M. Merriam L. A. May V. (2016). Activation of MEK/ERK signaling contributes to the PACAP-induced increase in guinea pig cardiac neuron excitability. *Am. J. Physiol. Cell Physiol.* 311 C643–C651. 10.1152/ajpcell.00164.2016 27488668 PMC5129752

[B51] Tu H. Liu J. Zhang D. Zheng H. Patel K. P. Cornish K. G. (2014). Heart failure-induced changes of voltage-gated Ca2+ channels and cell excitability in rat cardiac postganglionic neurons. *Am. J. Physiol. Cell Physiol.* 306 C132–C142. 10.1152/ajpcell.00223.2013 24025863 PMC3919990

[B52] Vaseghi M. Salavatian S. Rajendran P. S. Yagishita D. Woodward W. R. Hamon D. (2017). Parasympathetic dysfunction and antiarrhythmic effect of vagal nerve stimulation following myocardial infarction. *JCI Insight* 2:e86715. 10.1172/jci.insight.86715 28814663 PMC5621871

[B53] Vaseghi M. Shivkumar K. (2008). The role of the autonomic nervous system in sudden cardiac death. *Prog. Cardiovasc. Dis.* 50 404–419. 10.1016/j.pcad.2008.01.003 18474284 PMC2752648

[B54] Wang H. J. Rozanski G. J. Zucker I. H. (2017). Cardiac sympathetic afferent reflex control of cardiac function in normal and chronic heart failure states. *J. Physiol.* 595 2519–2534. 10.1113/JP273764 28116751 PMC5390865

[B55] Weihe E. Schutz B. Hartschuh W. Anlauf M. Schafer M. K. Eiden L. E. (2005). Coexpression of cholinergic and noradrenergic phenotypes in human and nonhuman autonomic nervous system. *J. Comp. Neurol.* 492 370–379. 10.1002/cne.20745 16217790 PMC2593918

[B56] Whyte K. A. Hogg R. C. Dyavanapalli J. Harper A. A. Adams D. J. (2009). Reactive oxygen species modulate neuronal excitability in rat intrinsic cardiac ganglia. *Auton. Neurosci.* 150 45–52. 10.1016/j.autneu.2009.04.005 19442588 PMC2751833

[B57] Zahner M. R. Li D. P. Chen S. R. Pan H. L. (2003). Cardiac vanilloid receptor 1-expressing afferent nerves and their role in the cardiogenic sympathetic reflex in rats. *J. Physiol.* 551 515–523. 10.1113/jphysiol.2003.048207 12829722 PMC2343227

[B58] Zhang D. Tu H. Cao L. Zheng H. Muelleman R. L. Wadman M. C. (2018). Reduced N-Type Ca(2+) Channels in Atrioventricular Ganglion Neurons Are Involved in Ventricular Arrhythmogenesis. *J. Am. Heart Assoc.* 7:e007457. 10.1161/JAHA.117.007457 29335317 PMC5850164

[B59] Zipes D. P. Rubart M. (2006). Neural modulation of cardiac arrhythmias and sudden cardiac death. *Heart Rhythm* 3 108–113. 10.1016/j.hrthm.2005.09.021 16399065 PMC2566299

